# Dropped Head Syndrome Secondary to Danon Disease: A Case Report

**DOI:** 10.7759/cureus.41191

**Published:** 2023-06-30

**Authors:** Vivek Bhat, Ganaraja V Harikrishna, Hyndav Kumar, Suresha Kodapala

**Affiliations:** 1 Internal Medicine, St. John's Medical College, Bangalore, IND; 2 Neurology, Vydehi Institute of Medical Sciences & Research Centre, Bangalore, IND

**Keywords:** lamp-2 deficiency, x linked disease, lysosomal storage disorder, inherited myopathy, hypertrophic cardiomyopathy

## Abstract

Dropped head syndrome (DHS) is characterized by neck extensor muscle weakness, which may be isolated or secondary to another neurologic diagnosis. DHS, due to lysosomal storage disorders, has not been reported in the literature. We present a 21-year-old male who had complaints of slowly worsening difficulty swallowing for the past eight years, along with difficulty keeping his head erect. His past medical history was significant for apical hypertrophic cardiomyopathy (HCM), and he had a history of sudden cardiac death in his immediate family. Clinical examination was significant only for neck extensor muscle weakness. His laboratory investigations were unremarkable, save for a significantly elevated creatine kinase (CK). Finally, whole exome sequencing identified a hemizygous stop gain variant in the lysosome-associated membrane protein 2 (LAMP-2) gene, pointing to a diagnosis of Danon disease (DD). DD is a rare, X-linked, inherited disease, due to a defect in the LAMP-2 gene that disrupts lysosomal autophagy. It is characterized by a triad of HCM, skeletal myopathy, and intellectual disability. Males typically suffer a more severe phenotype, and the cardiac disease drives its prognosis. Management involves regular cardiac monitoring, with appropriate physical therapy for myopathy and multidisciplinary treatment for intellectual disability. We suggest that DD be considered in the differential diagnosis for patients with HCM and elevated CK.

## Introduction

Dropped head syndrome (DHS) is characterized by weakness of the neck extensor muscles with or without concomitant neck flexor weakness. Usually, it affects elderly patients. Common etiologies include isolated neck extensor myopathy, Parkinson’s disease, myasthenia gravis, and amyotrophic lateral sclerosis [1]. DHS, due to glycogen or lysosomal storage disorders, has not been reported. We report a young adult male who presented to us with DHS, due to Danon disease (DD), a rare lysosomal storage disorder.

## Case presentation

Our patient was a 21-year-old male who presented with complaints of difficulty swallowing for the past eight years, which had slowly worsened, particularly over the last three years. The difficulty was initially with liquids, which progressed to include solids. Additionally, he had difficulty keeping his head raised and now had to bend his neck backward to facilitate swallowing. There was no diurnal variation, nor was there associated pain, regurgitation, difficulty chewing, or difficulty forming a bolus of food. There was no associated proximal or distal muscular, ocular, or bulbar weakness. He was born of a non-consanguineous union, had a normal birth and developmental history, and reported normal scholastic performance and behavior.

His family history was significant for the sudden cardiac death of his father at 52 years of age. His past history was significant for palpitations five years ago, for which he underwent cardiac evaluation at an outside center. Their echocardiogram revealed apical hypertrophic cardiomyopathy (HCM), and 24-hour Holter monitoring showed atrial premature beats, with no significant ventricular arrhythmias. Finally, their cardiac magnetic resonance imaging (MRI) reported evidence of non-specific infiltrative cardiomyopathy, with concentric hypertrophy, and a maximal wall thickness of 22 mm. A differential diagnosis of Fabry’s disease was suggested; however, the blood spot test for alpha-galactosidase enzyme was normal at 3.11 nmol/hr/ml (normal = 3-20 nmol/hr/ml).

**Figure 1:**
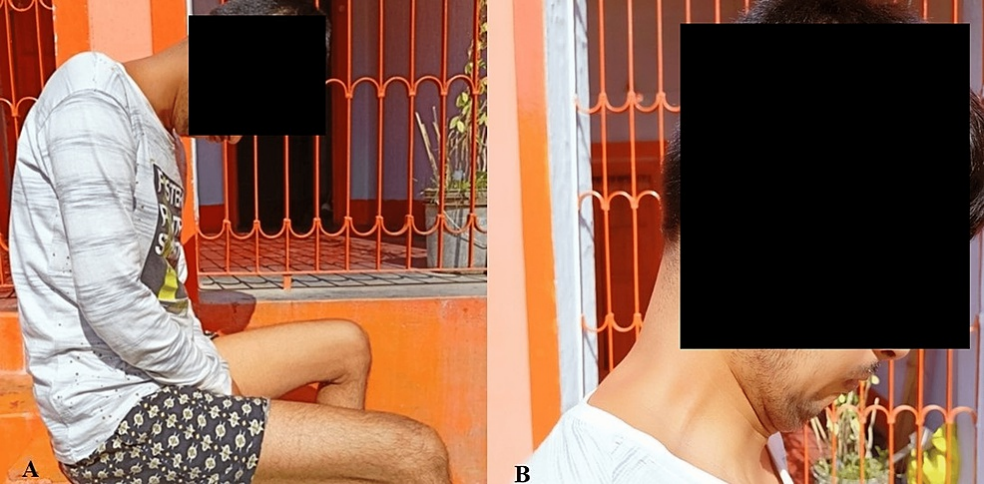
Patient demonstrating (A) dropped head posture at rest and (B) weakness of neck extensors.

His vital signs were within normal limits. On neurologic examination, he had normal cognition with no cranial nerve involvement. He had weakness of the neck extensors (Figure 1), but had normal power in the neck flexors and limbs. The rest of his neurologic examination was unremarkable.

His muscle MRI showed minimal signal changes in the neck muscles. Upper gastrointestinal endoscopy showed evidence of esophagitis and erosive gastroduodenitis, ruling out mechanical obstruction. His complete hemogram and basic metabolic panel were within normal limits. His creatine kinase (CK) levels were significantly elevated at 1218 U/L (normal = <170 U/L). Viral and autoimmune markers were negative. Finally, genetic studies with whole exome sequencing identified a hemizygous stop gain variant (c.C329C>A: p.S110X chrX:120455425G>T) in the lysosome-associated membrane protein 2 (LAMP-2) gene, confirming the diagnosis of DD. He was offered swallowing therapy, referred for genetic counseling, and advised regular follow-up for his HCM.

## Discussion

DD is incredibly rare, although it is being increasingly reported from all over the globe, likely due to the increased usage of genetic testing. When first reported in 1981 [2], it was originally classified as glycogen storage disease type-IIB [3]. However, it was later found that DD occurred due to a defect in the LAMP-2 gene and subsequent loss of the LAMP-2 protein, affecting lysosomal autophagy [4].

DD is inherited in an X-linked dominant manner [4] and is classically characterized by the triad of HCM, skeletal myopathy, and intellectual disability [3]. Males usually have a more severe phenotype, with early presentation and multisystem involvement, compared to females, who usually present later, and may have cardiac disease alone [3]. This is consistent with our case, who suffered from palpitations in his second decade of life itself. While skeletal muscle involvement is almost universal in male patients, it is usually mild [3]. Dysphagia is uncommon, and to our knowledge, our case is the first reported case of DHS due to DD. 

Management of DHS first hinges on appropriate diagnosis. At presentation, the workup includes CK, erythrocyte sedimentation rate/C-reactive protein, anti-acetylcholinesterase antibodies, muscle-specific kinase antibody titers, neck electromyography, and MRI of the cervical spine with contrast [1]. The definitive diagnosis, however, requires the identification of a LAMP-2 mutation, several of which have been described [3]. To our knowledge, our patient’s LAMP-2 mutation is a novel one. Once the diagnosis of DD is established, physical therapy and exercise constitute recommended therapy for DHS, as with other neuromuscular manifestations of DHS [3,5].

Pathologically, DD is characterized by intracytoplasmic vacuoles in the skeletal and cardiac muscles, explaining its earlier classification as and differential diagnosis of glycogen storage disorders [3]. Another differential with similar pathologic findings is X-linked recessive myopathy with excessive autophagy (XMEA). However, XMEA, which also has proximal muscle weakness, lacks other systemic features of DD [6].

The prognosis of DD is dependent on cardiac disease. Given that DD has been shown to represent 1-6% of patients with HCM or left ventricular hypertrophy, it is possible that DD is underdiagnosed [7,8]. Elevated CK is a clue to diagnosis, being seen in over 90% [3]. Management involves regular evaluation and potentially early transplantation for those with significant cardiomyopathy. Those with symptomatic arrhythmias may benefit from implantable cardioverter-defibrillator implantation [5].

## Conclusions

We report a patient with DD presenting as DHS. The diagnosis was established via the detection of the causative, novel LAMP-2 mutation. Appropriate supportive care was provided, and cardiac monitoring was recommended. Our case highlights a hitherto unreported presentation of DD and adds to the literature on this rare genetic syndrome.
